# Supplementary material for: The Effect of Traumatic Brain Injury on Sleep Architecture and Circadian Rhythms in Mice—A Comparison of High-Frequency Head Impact and Controlled Cortical Injury
Source: Biology (Basel). 2022 Jul 8;11(7):1031. doi: 10.3390/biology11071031 (PMC9312487; doi:10.3390/biology11071031)
Supplement: Supplementary file 1 [file biology-11-01031-s001.zip › biology-1726327-supplementary.pdf]

Time Period	Brain Wave	Sleep State	Sham (n)	CCI (n)	t-value, df	P=value
Dark Cycle	delta	wake	0.02217 ± 0.01169 (8)	0.01316 ± 0.002384 (12)	t=0.9134 df=18	0.3731
		NREM	0.002402 ± 0.0006183 (8)	0.00189 ± 0.0003241 (12)	t=0.8007 df=18	0.4338
		REM	0.0005798 ± 0.000161 (8)	0.0004583 ± 6.638e-005 (12)	t=0.7921 df=18	0.4386
	theta	wake	0.002185 ± 0.0006876 (8)	0.0016 ± 0.0002184 (12)	t=0.9496 df=18	0.3549
		NREM	0.000687 ± 0.0001579 (8)	0.0006026 ± 8.628e-005 (12)	t=0.5088 df=18	0.6171
		REM	0.0005493 ± 0.0001111 (8)	0.0006211 ± 0.0001368 (12)	t=0.3756 df=18	0.7116
	alpha	wake	0.001167 ± 0.0004324 (8)	0.0007853 ± 0.0001194 (12)	t=1.011 df=18	0.3256
		NREM	0.000592 ± 0.0001295 (8)	0.0004782 ± 7.699e-005 (12)	t=0.8064 df=18	0.4305
		REM	0.0003724 ± 7.615e-005 (8)	0.000411 ± 9.875e-005 (12)	t=0.2833 df=18	0.7802
	beta	wake	0.001306 ± 0.0004417 (8)	0.0009533 ± 0.0001486 (12)	t=0.8798 df=18	0.3906
		NREM	0.0004663 ± 0.0001002 (8)	0.0003766 ± 5.013e-005 (12)	t=0.8819 df=18	0.3895
		REM	0.0002796 ± 5.932e-005 (8)	0.0002603 ± 5.008e-005 (12)	t=0.2469 df=18	0.8078
	gamma	wake	0.000232 ± 5.68e-005 (8)	0.0001661 ± 2.166e-005 (12)	t=1.244 df=18	0.2294
		NREM	5.656e-005 ± 1.027e-005 (8)	4.847e-005 ± 6.351e-006 (12)	t=0.71 df=18	0.4868
		REM	5.8e-005 ± 1.171e-005 (8)	5.019e-005 ± 7.435e-006 (12)	t=0.5929 df=18	0.5606

Table S1a. The effect of HFHI on sleep microarchitecture in the dark cycle

Time Period	Brain Wave	Sleep State	Sham (n)	CCI (n)	t-value, df	P=value
Light Cycle	delta	wake	0.01632 ± 0.01083 (8)	0.01393 ± 0.003205 (12)	t=0.2491 df=18	0.8061
		NREM	0.002234 ± 0.0004396 (8)	0.001481 ± 0.0001998 (12)	t=1.746 df=18	0.0979
		REM	0.0006023 ± 0.0001687 (8)	0.0004758 ± 7.307e-005 (12)	t=0.7752 df=18	0.4483
	theta	wake	0.001455 ± 0.0005873 (8)	0.001612 ± 0.0003075 (12)	t=0.2587 df=18	0.7988
		NREM	0.0005985 ± 0.0001306 (8)	0.0005073 ± 7.871e-005 (12)	t=0.6367 df=18	0.5323
		REM	0.0005115 ± 0.0001289 (8)	0.0005127 ± 0.0001291 (12)	t=0.006217 df=18	0.9951
	alpha	wake	0.000811 ± 0.0003656 (8)	0.0007711 ± 0.0001311 (12)	t=0.1188 df=18	0.9067
		NREM	0.000497 ± 0.0001112 (8)	0.0004125 ± 6.806e-005 (12)	t=0.6881 df=18	0.5002
		REM	0.0003211 ± 6.984e-005 (8)	0.0003077 ± 7.456e-005 (12)	t=0.1243 df=18	0.9025
	beta	wake	0.0009233 ± 0.0003819 (8)	0.0009476 ± 0.000175 (12)	t=0.06474 df=18	0.9491
		NREM	0.0003948 ± 8.427e-005 (8)	0.0003214 ± 4.81e-005 (12)	t=0.8128 df=18	0.4270
		REM	0.0002662 ± 6.601e-005 (8)	0.0002483 ± 4.62e-005 (12)	t=0.2289 df=18	0.8215
	gamma	wake	0.0001717 ± 4.757e-005 (8)	0.0001599 ± 2.316e-005 (12)	t=0.2454 df=18	0.8089
		NREM	5.209e-005 ± 9.965e-006 (8)	4.086e-005 ± 5.89e-006 (12)	t=1.037 df=18	0.3137
		REM	5.546e-005 ± 1.222e-005 (8)	4.751e-005 ± 7.157e-006 (12)	t=0.6009 df=18	0.5554

Table S1b. The effect of HFHI on sleep microarchitecture in the light cycle

Time Period	Brain Wave	Sleep State	Sham (n)	CCI (n)	t-value, df	P=value
Dark Cycle	**delta**	wake	0.01186 ± 0.004228 (8)	0.008045 ± 0.003066 (8)	t=0.7307 df=14	0.4770
		**NREM**	**0.002043 ± 0.0003782 (8)**	**0.0007701 ± 0.0002359 (8)**	**t=2.855 df=14**	**0.0127**
		REM	0.002043 ± 0.0003782 (8)	0.0002437 ± 4.984e-005 (8)	t=1.217 df=14	0.2439
	**theta**	wake	0.003142 ± 0.001291 (8)	0.001797 ± 0.0006097 (8)	t=0.9421 df=14	0.3621
		NREM	0.0007824 ± 0.0002853 (8)	0.0003694 ± 0.0001558 (8)	t=1.271 df=14	0.2246
		**REM**	**0.0004975 ± 0.0001149 (8)**	**0.0002048 ± 4.37e-005 (8)**	**t=2.381 df=14**	**0.0320**
	alpha	wake	0.0008805 ± 0.0002216 (8)	0.00177 ± 0.001198 (8)	t=0.7297 df=14	0.4776
		NREM	0.0003989 ± 8.736e-005 (8)	0.0005274 ± 0.0003485 (8)	t=0.3577 df=14	0.7259
		REM	0.0003041 ± 5.768e-005 (8)	0.00029 ± 0.0001834 (8)	t=0.07335 df=14	0.9426
	beta	wake	0.001086 ± 0.0003336 (8)	0.0007034 ± 0.0001813 (8)	t=1.007 df=14	0.3308
		NREM	0.0003246 ± 6.817e-005 (8)	0.0001821 ± 2.452e-005 (8)	t=1.967 df=14	0.0693
		REM	0.000244 ± 5.643e-005 (8)	0.0001335 ± 1.871e-005 (8)	t=1.858 df=14	0.0843
	gamma	wake	0.0001809 ± 4.655e-005 (8)	0.0001759 ± 5.108e-005 (8)	t=0.07253 df=14	0.9432
		NREM	4.796e-005 ± 9.015e-006 (8)	5.08e-005 ± 2.287e-005 (8)	t=0.1154 df=14	0.9098
		REM	4.843e-005 ± 5.68e-006 (8)	4.961e-005 ± 2.143e-005 (8)	t=0.05357 df=14	0.9580

Table S2a. The effect of CCI on sleep microarchitecture in the dark cycle

Time Period	Brain Wave	Sleep State	Sham (n)	CCI (n)	t-value, df	P=value
Light Cycle	delta	wake	0.008811 ± 0.002857 (8)	0.007017 ± 0.003017 (8)	t=0.4318 df=14	0.6724
Light Cycle	delta	NREM	0.00143 ± 0.0003448 (8)	0.001053 ± 0.0003603 (8)	t=0.757 df=14	0.4616
Light Cycle	delta	REM	0.0004076 ± 9.539e-005 (8)	0.0004775 ± 0.0001878 (8)	t=0.3318 df=14	0.7450
Light Cycle	theta	wake	0.002021 ± 0.0006415 (8)	0.001954 ± 0.0007677 (8)	t=0.06672 df=14	0.9477
Light Cycle	theta	NREM	0.0006481 ± 0.0002301 (8)	0.0003813 ± 0.000125 (8)	t=1.019 df=14	0.3253
Light Cycle	theta	REM	0.0004558 ± 0.0001267 (8)	0.0002837 ± 6.195e-005 (8)	t=1.22 df=14	0.2427
Light Cycle	alpha	wake	0.0006914 ± 0.0001723 (8)	0.001946 ± 0.00143 (8)	t=0.8711 df=14	0.3984
Light Cycle	alpha	NREM	0.0003448 ± 8.414e-005 (8)	0.0004616 ± 0.0002865 (8)	t=0.3914 df=14	0.7014
Light Cycle	alpha	REM	0.0002683 ± 6.743e-005 (8)	0.0003181 ± 0.0002068 (8)	t=0.2292 df=14	0.8220
Light Cycle	beta	wake	0.0008188 ± 0.0002364 (8)	0.000697 ± 0.000222 (8)	t=0.3755 df=14	0.7129
Light Cycle	beta	NREM	0.0002785 ± 6.332e-005 (8)	0.0002001 ± 2.918e-005 (8)	t=1.124 df=14	0.2798
Light Cycle	beta	REM	0.0002139 ± 4.29e-005 (8)	0.0001801 ± 4.169e-005 (8)	t=0.565 df=14	0.5810
Light Cycle	gamma	wake	0.0001414 ± 3.277e-005 (8)	0.0001811 ± 6.4e-005 (8)	t=0.5521 df=14	0.5896
Light Cycle	gamma	NREM	4.131e-005 ± 6.704e-006 (8)	5.334e-005 ± 2.171e-005 (8)	t=0.5292 df=14	0.6050
Light Cycle	gamma	REM	5.201e-005 ± 1.071e-005 (8)	5.926e-005 ± 2.474e-005 (8)	t=0.269 df=14	0.7919

Table S2b. The effect of CCI on sleep microarchitecture in the dark cycle
